# Cavernous hemangioma of the frontal bone: a case report

**DOI:** 10.1186/1752-1947-8-121

**Published:** 2014-04-09

**Authors:** Domenico Murrone, Danilo De Paulis, Daniele F Millimaggi, Mattia Del Maestro, Renato J Galzio

**Affiliations:** 1 Department of Neurosurgery, San Salvatore City Hospital, via Vetoio, Coppito, L’Aquila 67100, Italy; 2 Department of Life, Health and Environmental Sciences, University of L’Aquila, Piazza S. Tommasi, Coppito, L’Aquila 67100, Italy

**Keywords:** Cavernous hemangioma, Cranial bone hemangioma, Cranioplasty

## Abstract

**Introduction:**

Cavernous hemangiomas are rare benign bone tumors and those at the level of the cranial bones are even rarer.

**Case presentation:**

A 50-year-old woman of Italian ethnicity presented with a frontal mass. A computed tomography scan showed an osteolytic lesion and a magnetic resonance imaging scan revealed a hypointense lesion on the T1-weighted image and a hyperintense lesion on the T2-weighted image. We performed a tailored craniectomy and cranioplasty. Histological examination revealed a cavernous hemangioma.

**Conclusions:**

These benign tumors do not have classic radiographic features and so can be misinterpreted as lesions like multiple myeloma or osteosarcoma. Consequently, the diagnosis is most often made during surgical resection.

## Introduction

Cavernous hemangiomas are uncommon tumors, accounting for 0.7 to 1 percent of all bone neoplasms [1]. These tumors, arising from the intrinsic vasculature of the bone, are mostly found in vertebral bodies and represent 0.2 percent of all benign neoplasms of the skull. The majority of these lesions are asymptomatic, but patients can present with focal pain or a palpable mass [2].

## Case presentation

**Figure 1:**
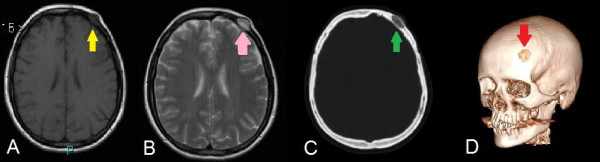
A magnetic resonance imaging scan shows a hypointense lesion (yellow arrow) on the T1-weighted image (A) and a hyperintense lesion (pink arrow) on the T2-weighted image (B); a computed tomography scan reveals an osteolytic lesion (green arrow) with erosion of the tabula externa (C); a three-dimensional reconstruction of the skull with evidence of the lesion in the pre-operative time (red arrow) (D).

We present the case of a 50-year-old woman of Italian ethnicity who presented with a slow-growing frontal mass, tender to pressure, with spontaneous pain. Her neurological examination was completely normal. There was no history of trauma or other systemic disease. A magnetic resonance imaging (MRI) scan showed a hypointense lesion on the T1-weighted image and a hyperintense lesion on the T2-weighted image. A computed tomography (CT) scan showed an osteolytic lesion with erosion of the tabula externa (Figure 1). A left frontal craniectomy via linear incision was performed with excision of the frontal lesion and a margin of surrounding uninvolved bone and a cranioplasty. There was no involvement of the underlying dura. This lesion was well delineated and brown in color with erosion of the outer tables of the skull. Her postoperative course was uneventful and a CT scan showed complete resection of the mass with a correct cranioplasty. A histological examination revealed a cavernous hemangioma of the diploe with large, thin-walled, dilated capillary spaces lined by flattened endothelial cells without evidence of malignancy.

## Discussion

The most commonly involved site of intraosseus hemangiomas is the vertebrae, and hemangiomas of the calvarial bones account for 0.2 percent of all bone neoplasms [1,3]. Women are two times more commonly afflicted than are men and the peak age incidence is the fourth decade [4], although pediatric cases are described [5]. The frontal bones are the most commonly affected, followed by the parietal bones [3]. A skull hemangioma occurring at the site of a cranioplasty was described [6]. The great majority of reported cases of hemangiomas are unifocal but multiple hemangiomas have been reported [4]. Trauma seems not to be a predisposing factor in the development of these lesions. Hemangiomas may be the result of faulty differentiation of primordial vessels, resulting in an abnormal capillary bed. Hemangiomas have been classified as cavernous, that is predominant in hemangiomas of the skull, capillary, or venous [7]. In most calvarial hemangiomas, the inner table remains intact, allowing *en bloc* resection. Plain radiography or CT scan reveals this lesion as solitary lytic lesions with a sclerotic rim while MRI shows isointense on T1-weighted images and hyperintense on T2-weighted images, consistent with regions of slow-flowing blood. Sometimes, the classic radiographic appearances are not evident. Consequently, the diagnosis is most often made during surgical resection. These tumors can be misinterpreted as lesions like multiple myeloma or osteosarcoma [8]. The first report of a primary hemangioma of the skull was by Toynbee [9] in 1845 and the most important review was published by Heckl [3] in 2002 regarding 103 histologically proven cases between 1975 and 2000. The gold standard treatment is *en bloc* resection of the tumor with the removal of a rim of normal bone, described in 1923 by Cushing [10]. Instead, radiotherapy should be reserved for those few cases that are not safely resectable [11]. Cases of recurrence of this lesion after complete resection have not been described.

## Conclusions

Calvarian hemangiomas do not always have typical radiologic features and should always be considered in the differential diagnosis of malignant skull lesions. Histopathological confirmation after surgical resection of the tumor is the definitive method for diagnosis.

## Consent

Written informed consent was obtained from the patient for publication of this case report and any accompanying images. A copy of the written consent is available for review by the Editor-in-Chief of this journal.

## Abbreviations

CT: computed tomography; MRI: magnetic resonance imaging.

## Competing interests

The authors declare they have no competing interests.

## Authors’ contributions

All authors analyzed and interpreted the patient data and contributed to writing the manuscript. All authors read and approved the final manuscript.
